# Clustered Regularly Interspaced Short Palindromic Repeat/Cas12a Mediated Multiplexable and Portable Detection Platform for GII Genotype Porcine Epidemic Diarrhoea Virus Rapid Diagnosis

**DOI:** 10.3389/fmicb.2022.920801

**Published:** 2022-06-09

**Authors:** Bingxu Qian, Kai Liao, Dexin Zeng, Wanqing Peng, Xiaodong Wu, Jinming Li, Zongyi Bo, Yongxin Hu, Wenlong Nan, Yuan Wen, Yuying Cao, Feng Xue, Xiaorong Zhang, Jianjun Dai

**Affiliations:** ^1^MOE Joint International Research Laboratory of Animal Health and Food Safety, College of Veterinary Medicine, Nanjing Agricultural University, Nanjing, China; ^2^National Research Center for Exotic Animal Diseases, China Animal Health and Epidemiology Center, Qingdao, China; ^3^Technology Center of Hefei Customs, Hefei, China; ^4^Joint International Research Laboratory of Agriculture and Agri-Product Safety, The Ministry of Education of China, Yangzhou University, Yangzhou, China; ^5^Jiangsu Co-Innovation Center for the Prevention and Control of Animal Infectious Disease and Zoonoses, College of Veterinary Medicine, Yangzhou University, Yangzhou, China; ^6^School of Life Science and Technology, China Pharmaceutical University, Nanjing, China

**Keywords:** porcine epidemic diarrhoea virus, GII genotype, CRISPR/Cas12a, rapid diagnosis, lateral flow strip

## Abstract

Porcine epidemic diarrhoea virus (PEDV) is a member of the genus *Alphacoronavirus* in the family *Coronaviridae*. It causes acute watery diarrhoea and vomiting in piglets with high a mortality rate. Currently, the GII genotype, PEDV, possesses a high separation rate in wild strains and is usually reported in immunity failure cases, which indicates a need for a portable and sensitive detection method. Here, reverse transcription–recombinase aided amplification (RT-RAA) was combined with the Clustered Regularly Interspaced Short Palindromic Repeat (CRISPR)/Cas12a system to establish a multiplexable, rapid and portable detection platform for PEDV. The CRISPR RNA (crRNA) against Spike (S) gene of GII PEDV specifically were added into the protocol. This system is suitable for different experimental conditions, including ultra-sensitive fluorescence, visual, UV light, or flow strip detection. Moreover, it exhibits high sensitivity and specificity and can detect at least 100 copies of the target gene in each reaction. The CRISPR/Cas12a detection platform requires less time and represents a rapid, reliable and practical tool for the rapid diagnosis of GII genotype PEDV.

## Introduction

Coronaviruses (CoVs) are a type of enveloped virus consisting of a positive-strand RNA genome and includes numerous viruses of veterinary and human importance (Su et al., 2016). SARS, MERS, and COVID-19 have claimed tens of millions of lives and have had a profound health and economic impact on human society. As a member of the genus *Alphacoronavirus* of the family *Coronaviridae*, the porcine epidemic diarrhoea virus (PEDV), causes acute intestinal infectious diseases, which manifest as severe dehydration, diarrhoea, vomiting, and a high mortality rate in piglets (Huang et al., 2013; Jung and Saif, 2015). PEDV was first reported in 1971 in the United Kingdom (Chasey and Cartwright, 1978). Seven years later, it was first observed by electron microscopy in the intestinal contents of piglets in European swine breeding farms. Currently, it is prevalent in Europe, Asia, and the United States and has raised global concerns (Huang et al., 2013; Jung and Saif, 2015; Song et al., 2015).

Porcine epidemic diarrhea virus may be divided into two different genotypes, GI and GII, according to multiple INDEL and point mutations on the S1 subunits of the spike (S) protein (Li et al., 2016). The widely used CV777 vaccine strain of the PEDV GI genotype induces a robust immune response in pigs and successfully has controlled classical PEDV infection (Li et al., 2017) for almost 2 decades (Li et al., 2012). However, after 2010, PEDV outbreaks caused by variant strains have occurred in vaccinated pigs (Li et al., 2012; Sun et al., 2012). Molecular epidemiological studies have shown that these variant strains isolated in China belongs to an independent branch of genetic evolution, designated the GII genotype, and the CV777-inactivated or live-attenuated vaccines do not provide adequate immune protection. Between 2013 and 2014, an outbreak of diarrhea was reported in swine herds in the United States. Genetic evolution analysis revealed that the strain was similar to the Chinese GII PEDV variant strain (Chen et al., 2014; Vlasova et al., 2014). Currently, the GII PEDV strain has become the major epidemic strain of PEDV and has caused significant economic losses for the pig industry.

For epidemic prevention and control of PEDV, rapid disease diagnosis is essential in addition to effective vaccines. PCR-based assays for the detection of PEDV are the preferred method because they have the advantages of simple operation, high sensitivity, and specificity (Kim et al., 2007). In addition to PCR, quantitative real-time PCR (qRT-PCR), a more sensitive and rapid assay, had also been widely used. Specific primers targeting the N gene sequence of PEDV have been used for real-time PCR assays based using SYBR Green І detection chemistry (Zheng et al., 2020). In general, the specificity and sensitivity of TaqMan probe real-time PCR methods are considered better than intercalating dye-based real-time PCR. A TaqMan probe-based real-time PCR assay targeting the ORF1a region of PDEV has shown high sensitivity and specificity, with a detection limit of 1 × 10^2^ copies/μl for PEDV (Pan et al., 2020). Moreover, a TaqMan probe-based real-time PCR assay that differentiates variant from classical PDEV was also developed with a limit of detection (LOD) of 5 × 10^2^ DNA copies (Zhao et al., 2014). Although qRT-PCR has high sensitivity and specificity, it relies on expensive instruments and professional technicians, which cannot fully meet the necessity of on-site detection.

Clustered regularly interspaced short palindromic repeat (CRISPR)-Cas systems are adaptive immune systems found in bacteria and archaea. The CRISPR system is based on CRISPR RNAs (crRNAs) that bind to CRISPR-associated (Cas) proteins to form a complex, which directly cleavages complementary sequences (Mali et al., 2013). CRISPR-Cas systems can be classified into Class 1 (Makarova et al., 2017a) and Class 2 (Makarova et al., 2017b), of which Class 1 systems require multiple effector protein complexes; whereas Class 2 systems require only a single effector protein to function (Shmakov et al., 2017). The relatively simple architecture of the effector complexes has made Class 2 systems a more popular option for use as a genome-editing tool (Jinek et al., 2012). The most widely used Class 2 systems include CRISPR/Cas9, CRISPR/Cas12a, and CRISPR/Cas13a. Given the strong specificity of the recognition sequence of CRISPR/CAS systems, one can expect to establish a more sensitive and effective platform for nucleic acid detection. In 2017, a platform termed specific high sensitivity enzymatic reporter unlocking (SHERLOCK) combined isothermal preamplification with Cas13 for the detection of single molecules of RNA or DNA (Gootenberg et al., 2017). Subsequently, four advances integrated into SHERLOCK version 2 (SHERLOCKv2) could detect Dengue or Zika virus using lateral flow strips (LFSs), which highlights its potential as a multiplexable, portable, rapid and quantitative detection platform for nucleic acids (Gootenberg et al., 2018). CRISPR/Cas12a systems unleash indiscriminate single-stranded deoxyribonuclease (ssDNase) cleavage activity that specifically cleave single-stranded DNA (ssDNA). The DNA Endonuclease Targeted CRISPR *Trans*-Reporter (ADETECTR) platform, which enables rapid and specific detection of human papillomavirus in patient samples, was established by combining Cas12a ssDNase activation with isothermal amplification (Chen et al., 2018).

The emergence and development of CRISPR enable new opportunities for the rapid and convenient diagnosis of viral diseases. Here, we report a molecular method for the detection of the dominant GII PEDV spike (S) gene using a CRISPR/Cas12a system, which is not limited by expensive instrumentation, skilled analysis, and complex processes. This platform relies on a fluorescent detection system (FDS) or two visual detection systems for signal sensing of the trans-cleavage activity of the Cas12a protein triggered by the spike (S) gene of PEDV. It can specifically distinguish GI and GII PEDV with high sensitivity and specificity, providing an effective detection tool for prevention and control of this virus.

## Materials and Methods

### Reagents and Instruments

The FastPure Cell/Tissue Total RNA Isolation Kit V2 (RC112-01), the HiScript® III 1st Strand cDNA Synthesis Kit (R312-01), and T7 High Efficiency Transcription Kit (JT101-01) were purchased from Vazyme Biotech Co. Ltd. (Nanjing, China). The Recombinase Aided Amplification (RAA) kit (WLB8201KIT) was purchased from Warbio Biotech Co. Ltd. (Nanjing, China). The EnGen™ LbCas12a (#M0653T) and NEBuffer™ 2.1 (#B7202S) were purchased from New England Biolabs (Ipswich, United States). RNase inhibitor and PerfectStart® II Probe qPCR SuperMix were purchased from TransGen Biotech Co. Ltd. (Beijing, China). Synthesis of the target sequence for positive control, crRNA, 12 nt oligo reporter, primer, and probe, as well as gene sequencing, was accomplished by Tsingke Biotech Co. Ltd. (Beijing, China). The Spark® multi-mode microplate reader was purchased from Tecan Trading Co. Ltd. (Pudong, China). Applied Biosystems StepOne™ Real-Time PCR System was purchased from Thermo Fisher Scientific Inc. (Waltham, United States).

### The Nucleic Acid of Viruses

The genomic RNA or DNA of Transmissible gastroenteritis virus (TGEV), Porcine deltacoronavirus (PDCoV), and Pseudorabies virus (PrV) were presented by Zhang Xiaorong, Associate Professor, College of Veterinary Medicine, Yangzhou University (Yangzhou, China). The genomic DNA of ASFV was presented by the National Research Center for Exotic Animal Diseases, Animal Health and Epidemiology Center (Qingdao, China). The virus strain Porcine circovirus (PCV) and Seneca virus A (SVA) were isolated from clinical samples in our laboratory.

### Clinical Samples

The clinical samples were all collected from a pig farm with an outbreak of PEDV. Rectal swabs were collected according to the standards recommended by the OIE.

### PEDV Target Design

S gene sequences of Genotype I and Genotype II PEDV were downloaded from the National Center for Biotechnology Information (NCBI) database. The target sequences were analyzed by SnapGene to design specific crRNAs and primers of Genotype II PEDV. These designed sequences were used to alignment by BLAST with the swine genome (taxid: 9823), PDCoV, transmissible gastroenteritis virus (TGEV), porcine respiratory coronavirus (PRCV), and the swine acute diarrhea syndrome coronavirus (SADS-CoV) for verifying the specificity. When CRISPR/Cas12a proteins were bound to double-stranded DNA (dsDNA) guided by specific crRNA, its trans-cleavage activity was nonspecifically activated to cleave ssDNA-FQ-reporter followed by signal sensor.

### Genomic RNA Extraction and Reverse Transcription From Clinical Samples

The rectal swabs (*n* = 72) were collected from the clinical samples of swine breeding farms with an outbreak of diarrhoeal. After three times of freezing and thawing, the supernatant of 200 μl was obtained by centrifugation. Subsequently, 30 μl of RNA were extracted from 200 μl supernatant of the clinical sample by the FastPure Cell/Tissue Total RNA Isolation Kit V2, and 1 μg of RNA was reverse transcribed into cDNA using HiScript® III 1st Strand cDNA Synthesis Kit.

### Plasmid and crRNA Preparation

The full-length S gene fragment of PEDV/JSX2014 (GenBank: MH056658.1) was amplified by the primers PEDV-S-F/R listed in Supplementary Table 1; pUC57-PEDV-S was constructed by recombining pUC57 with S gene through EcoR I and BamH I restriction site at 5′ and 3′ terminals. The pUC57 plasmids containing crRNAs (pUC57-crRNA) were synthesized by GenScript (Jiangsu, China). The names and sequences of crRNAs are listed in Supplementary Table 2. pUC57-crRNA was used as the template for transcription to obtain crRNA using T7 High Efficiency Transcription Kit (Vazyme Biotech Co. Ltd., Jiangsu, China). RNA products were aliquoted and stored at −80°C until usage.

### Optimization of CRISPR/Cas12a Fluorescent Detection System

In 100 μl of reaction system of CRISPR, the fluorescence signal activated by trans-cleavage in 96-well Tecan black flat was read by the Spark® multi-mode microplate reader, and the initial value which was favorable for observing the change of fluorescence signal was obtained. Firstly, the optimal quantity of ssDNA-FQ-reporter was confirmed with a molar gradient of 0.0625 pmolto 2 pmol. The name and sequence of reporter are listed in Supplementary Table 1. And then, the verified molar gradient of Cas12a was from 0.5 to 10 pmol. The CRISPR-FDS were performed under 15, 28, 37, and 42°C, respectively, for identifying optimal reaction temperature. After the optimal condition was determined, the visible fluorescence could be observed under UV light by increasing the concentration of the ssDNA-FQ-reporter.

### RAA Reactions and Primer Design

Recombinase aided amplification of the PEDV S gene was performed by the commercial RAA kit (Warbio biological Co., Ltd., Jiangsu, China), primers were designed by Primer 5.0 according to the manufacturer’s instructions. The PEDV RAA primers named PEDV-RAA-F/R listed in Supplementary Table 1 were designed according to the instructions. A typical 50 μl reaction system contained 2 μl of cDNA template, 2 μl of PEDV-RAA-F (forward primer, 10 mM), 2 μl of PEDV-RAA-R (reward primer, 10 μM), 29.4 μl of A Buffer, 2.5 μl of B Buffer, and 12.1 μl of ddH2O. The reaction tube was incubated at 37°C for 30 min for subsequent CRISPR/Cas12a cleavage reaction.

### Preparation of Lateral Flow Strips

**Figure 1:**
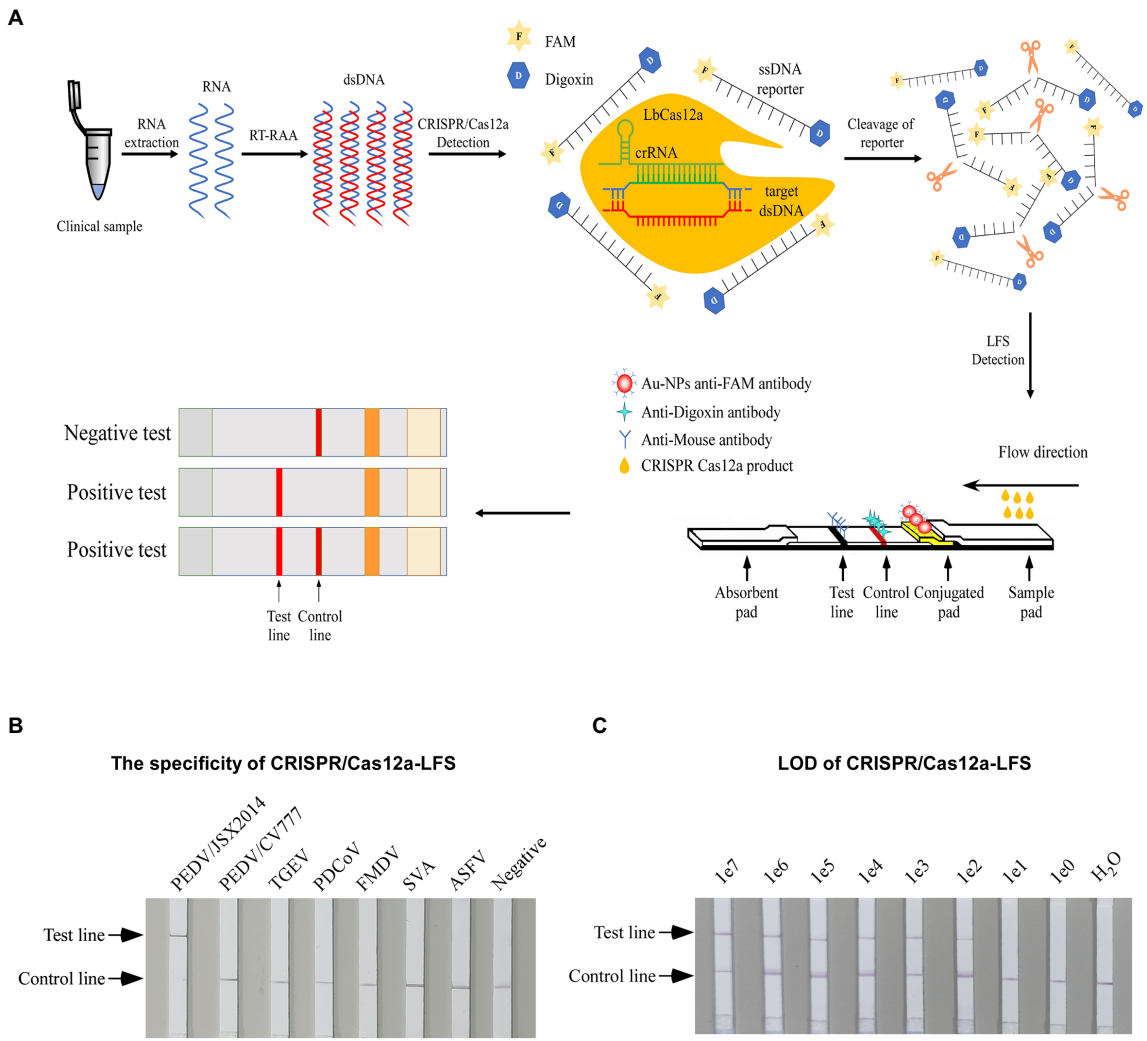
Establishing the CRISPR/Cas12a-LFS. **(A)** Schematic diagram of PEDV visual detection, the method combines RAA assay, the CRISPR/Cas12a-FDS, and lateral flow strips (LFSs). The ssDNA reporter was labeled with FAM and digoxin (ssDNA-FD-reporter) at the 5′ and 3′ termini, respectively. The immunochromatographic strip using Au-NPs anti-FAM antibody to show the readout. The sample band was only shown when the ssDNA-FD-reporter was cleaved by CRISPR/Cas12a, which is activated by PEDV cDNA. Both the control line and the test line showed when the ssDNA-FD-reporter was partially cleaved, and only the test line showed when the ssDNA-FD-reporter was completely cleaved. **(B)** The specificity of different porcine viruses was detected by CRISPR/Cas12a-LFS. **(C)** The LOD of PEDV S gene was detected by CRISPR/Cas12a-LFS. Serially diluted synthetic pUC57-PEDV-S was used as the template.

Preparation of gold nanoparticles by sodium citrate reduction method. Heat 0.01% chlorauric acid hydrate to a boil, then quickly add 1 ml 1% sodium citrate while stirring. Let the solution turn wine red and continue to boil for 15 min, then cool to room temperature. Transmission electron microscope was used to scan the ultrastructure of gold nanoparticles, and their aggregation state and particle size distribution were then observed. FAM antibody was coupled with gold nanoparticles and attached to the binding pad of the strip. Digoxin antibody and *rat* resistance were sprayed on NC film as quality control line and detection line. The principle of LFSs detection is shown in Figure 1A.

### Lateral Flow Strips Detection Reactions

In lateral flow detection, the reporter ssDNA probe sensor was labeled with FAM and digoxin at the 5′and 3′ termini, respectively. The sequence of ssDNA-FD-reporter is listed in Supplementary Table 1. The optimal CRISPR/Cas12a detection reaction followed the conditions determined by CRISPR/Cas2a-FDS, and the strips were then inserted into the reaction and incubated at room temperature for 3 min. The strips were then taken out and photographed using a camera.

### Real-Time PCR Detection

The TaqMan real-time PCR detection of the PEDV S gene was carried out using a StepOnePlus™ Real-Time PCR System (Applied Biosystems, Massachusetts, United States) according to the method previously reported. The TaqMan RT-PCR was performed in a final volume of 20 μl containing 10 μl of 2× AceQR qPCR Probe Master Mix (Vazyme Biotech Co., Ltd., Nanjing, China), 0.4 μl of primer PEDV-q-F, 0.4 μl of primer PEDV-q-R, 0.2 μl of TaqMan probe PEDV-probe, 2 μl of cDNA, and 7 μl of ddH2O. The PCR program was as follows: initial denaturation at 95°C for 5 min followed by 40 cycles of denaturation (95°C for 10 s), annealing, and extension (60°C for 30 s). The fluorescence signal collection occurred at the 60°C annealing extension per cycle. The cycle value (Ct) ≤ 35.0 was judged as PEDV positive. The sequence of the primers and probe used for qRT-PCR are listed in Supplementary Table 1.

### Statistical Analysis

All experiments were performed in triplicate, and data were shown as the mean ± SD. Statistical analysis and graphing were carried out with GraphPad Prism 8.0.

### Data Availability

All data that support the findings of this study are available from the corresponding author upon reasonable request.

## Results

### Detection of PEDV cDNA With CRISPR/Cas12a Detector

**Figure 2:**
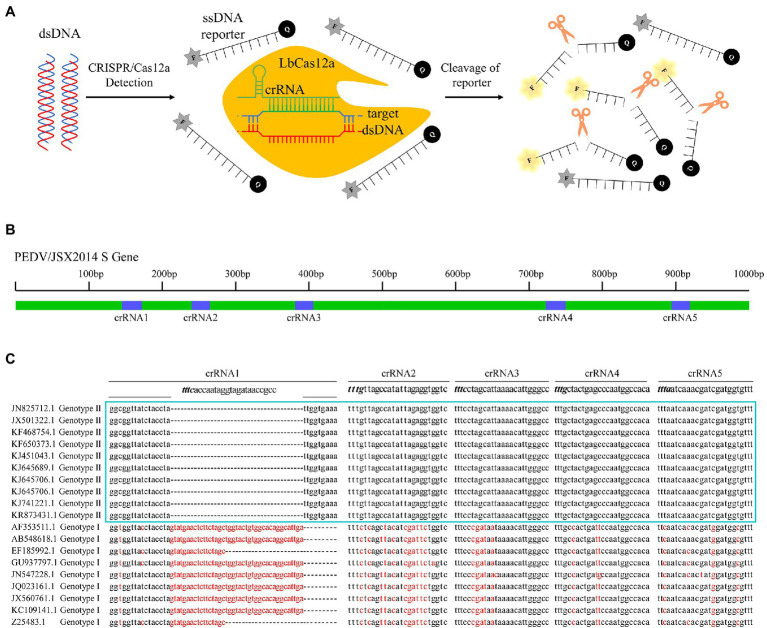
Design of clustered regularly interspaced short palindromic repeat (CRISPR) RNA (crRNA) of porcine epidemic diarrhoea virus (PEDV) Spike gene and schematic diagram of CRISPR/Cas12a-fluorescent detection system (FDS). **(A)** Schematic diagram of the CRISPR/Cas12a-FDS assay. Specific crRNAs targeting the PEDV Spike gene were designed for PEDV genome detection. When CRISPR/Cas12a proteins were bound to double-stranded DNA (dsDNA) guided by specific crRNA, it nonspecifically activated its trans-cleavage activity, the quenched fluorescent ssDNA was cleaved and thus stimulating fluorescence. F, fluorophore; Q, quencher. **(B)** Five crRNAs targeting Spike gene selected for PEDV detection and the relative positions of these crRNAs in S gene. **(C)** Sequence alignment of Spike genes from 19 strains of PEDV targeted by crRNAs. The nucleotide variants from the consensus sequence were highlighted with red color.

To sensitively and specifically detect PEDV genomic cDNA, a CRISPR/Cas12a fluorescence detection system (CRISPR/Cas12a-FDS) was established consisting of the LbCas12a protein, PEDV-specific CRISPR RNAs, and an ssDNA-FQ-reporter (Figure 2A). As CRISPR/Cas12a recognizes a T (thymine) nucleotide-rich protospacer-adjacent motif (PAM), five crRNAs (crRNA1 ~ crRNA5) with a TTTN PAM targeting the S gene of the Genotype II PEDV were designed (Figure 2B). These crRNA-specific binding sequences contained multiple insertions and mutations of the Genotype I PEDV (Figure 2C).

### The Optimization of CRISPR/Cas12a-FDS

**Figure 3:**
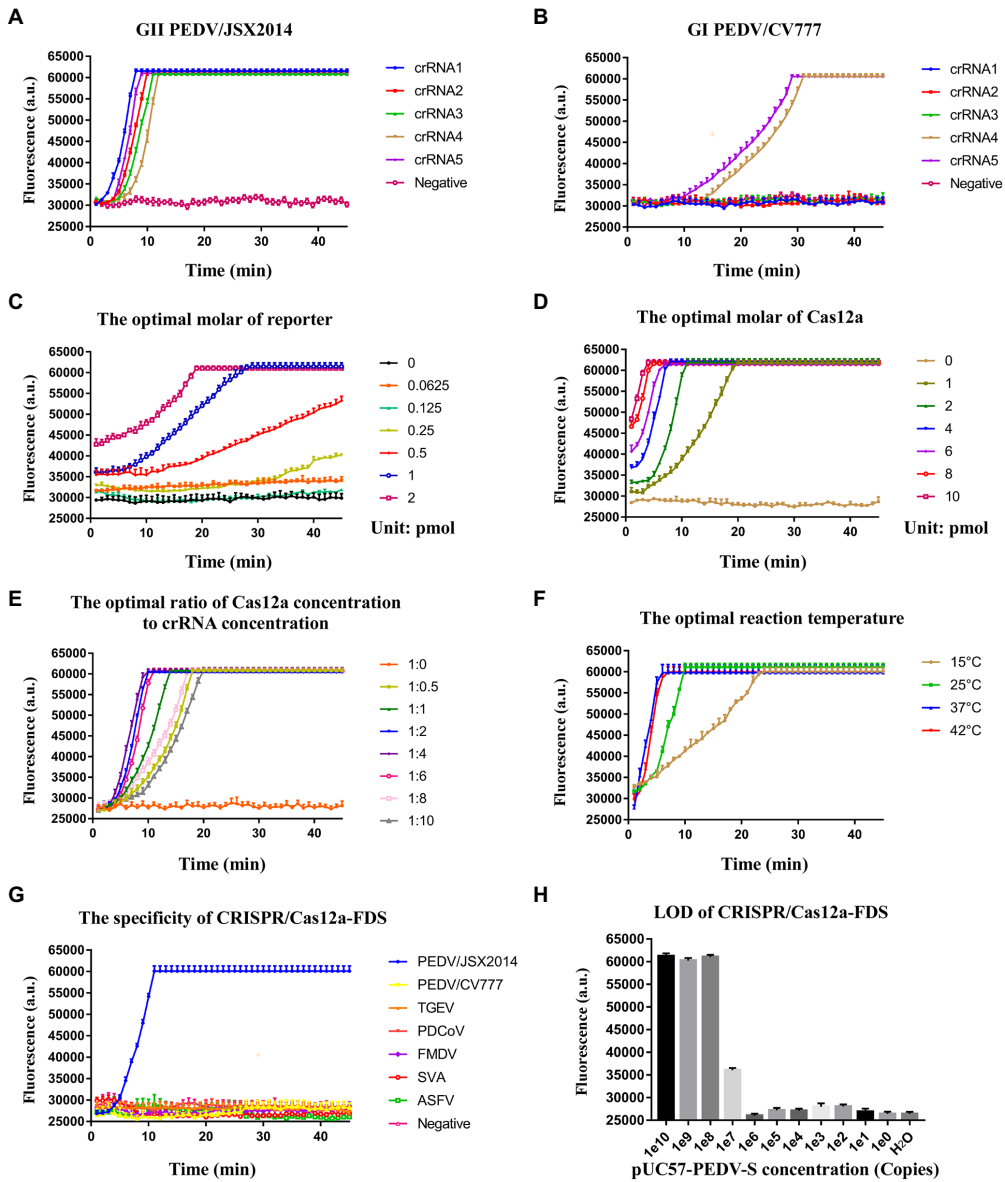
The optimization of the CRISPR/Cas12a-FDS assay. **(A)** The time course of GII PEDV/JSX2014 was detected by five crRNAs (crRNA1–crRNA5) respectively in CRISPR/Cas12a-FDS. **(B)** The time course of GI PEDV/CV777 was detected by five crRNAs (crRNA1–crRNA5) in CRISPR/Cas12a-FDS. **(C)** The time course of the GII PEDV/JSX2014 genome was detected by CRISPR/Cas12a-FDS when the molar range of ssDNA-FQ reporter was 0–2 pmol. **(D)** The time course of the GII PEDV/JSX2014 genome was detected by CRISPR/Cas12a-FDS when the molar range of Cas12a protein was 0–10 pmol. **(E)** The time course of the GII PEDV/JSX2014 genome was detected by CRISPR/Cas12a-FDS when the ratio of Cas12a concentration to crRNA concentration ranged from 1:0 to 1:10. **(F)** The time course of the GII PEDV/JSX2014 genome was detected by CRISPR/Cas12a-FDS when the temperature of reaction ranged from 15 to 42°C. Error bars in panels **(A–F)** represent the mean ± SD, where *n* = 3 replicates. **(G)** The cDNA of PEDV detection with CRISPR/Cas12a-FDS at 37°C in 45 min. No fluorescent amplification was detected for the nucleic acids of other tested porcine viruses, GI PEDV, TGEV, PDCoV, FMDV, SVA, and ASFV. **(H)** Sensitivity of the CRISPR/Cas12a-FDS. The serially diluted pUC57-PEDV-S plasmid was used as a template.

To determine whether the crRNAs could specifically distinguish GI and GII PEDV strains, the genomes of PEDV/JSX2014 (GII) and PEDV/CV777 (GI) were used as templates for CRISPR/Cas12a-FDS detection. All of the crRNAs bound specifically to the genomic template of GII PEDV; whereas crRNA4 and crRNA5 bound non-specifically to the GI PEDV genome template (Figures 3A,B). The crRNA1 showed better amplification efficiency compared with the other crRNAs (Figure 3A). Therefore, crRNA used in subsequent experiments were crRNA1. To achieve the best detection performance, we optimized the molarity of the ssDNA reporter and Cas12a, the concentration ratio of Cas12:crRNA, and the reaction temperature by measuring the dynamics of fluorescence intensity (Figures 3C–F). The fluorescence amplification could not be detected when the molarity of the reporter was below 0.25 pmol and the amplification efficiency was the highest when 1 pmol of the reporter was used (Figure 3C). With an increase in the molarity of Cas12a, the time for CRISPR/Cas12a-FDS to reach maximum fluorescence was reduced. Considering the amplification efficiency and background fluorescence, the optimal content of Cas12a was 2 pmol (Figure 3D). When the concentration ratio of Cas12a to crRNA was in the range of 1:0.5–1:4, the amplification efficiency gradually increased (Figure 3E). When it surpassed 1:4, the amplification efficiency decreased (Figure 3E). Finally, the amplification efficiency at 15 and 25°C was significantly lower compare with that at 37 and 42°C, whereas the reaction temperature between 37 and 42°C did not show a significant difference for amplification efficiency (Figure 3F). Moreover, CRISPR/Cas12a-FDS showed no cross-reactivity with other tested swine viruses including the double-stranded DNA virus, ASFV (Figure 3G). The LOD for CRISPR/Cas12a-FDS was 10^8^ copies (Figure 3H).

### Establishment of Visualized RAA-CRISPR-FDS

**Figure 4:**
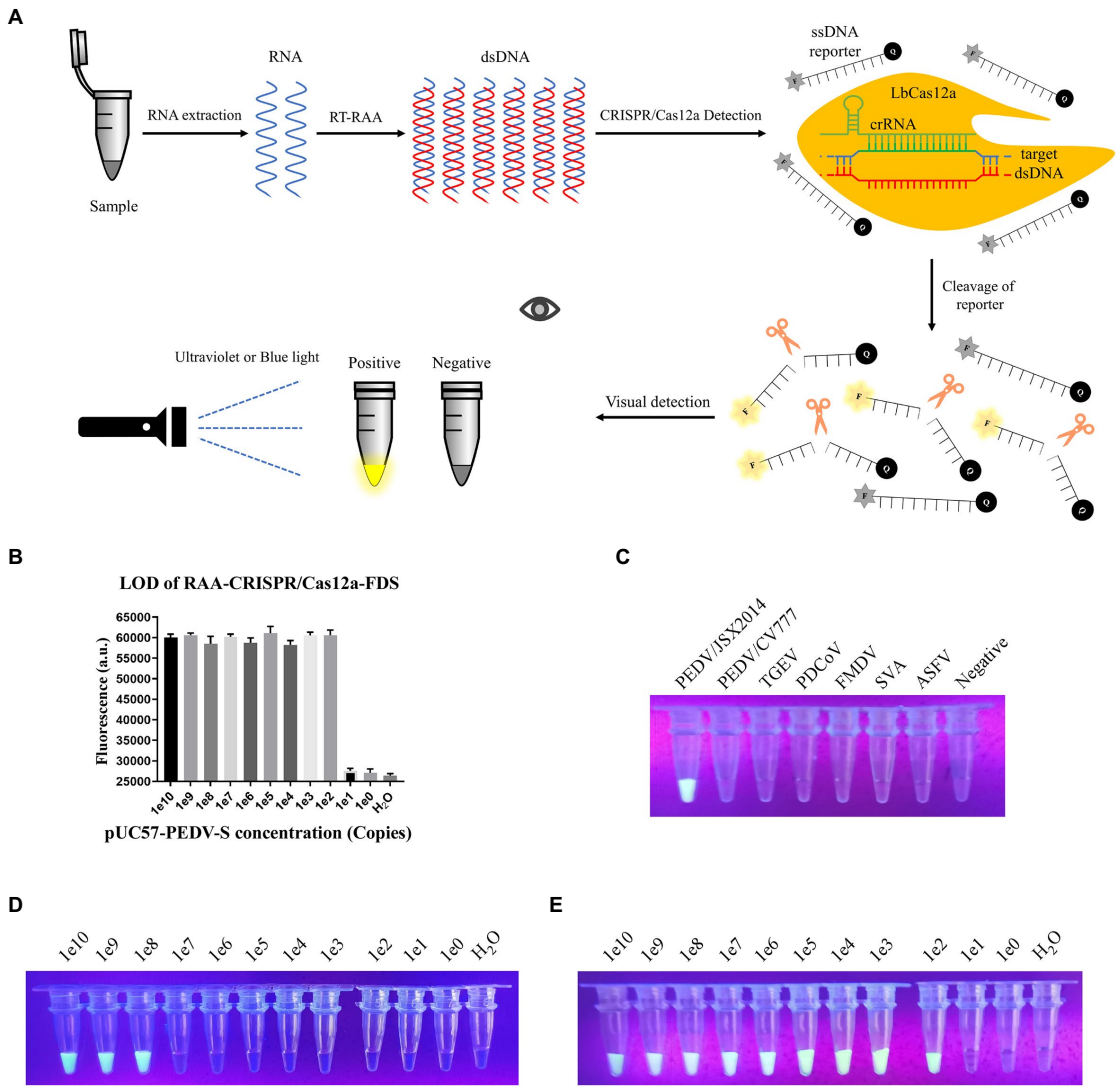
Establishing the Recombinase Aided Amplification (RAA)-CRISPR-FDS. **(A)** Schematic diagram of PEDV visual detection, the method combines RAA assay, and the CRISPR/Cas12a-FDS. **(B)** Sensitivity of the CRISPR/Cas12a-FDS combined with RAA. The serially diluted pUC57-PEDV-S plasmid was used as a template. **(C)** After samples were detected by CRISPR/Cas12a-FDS at 37°C, the fluorescence of different porcine viruses was observed under UV light by the gel imaging system at the 30th min. Fluorescence could only be observed in PEDV/JSX2014 tubes. **(D)** Sensitivity of the CRISPR/Cas12a-FDS visual observations. The serially diluted pUC57-PEDV-S plasmid was used as a template. **(E)** Sensitivity of the CRISPR/Cas12a-FDS combined with RAA visual observations. The serially diluted pUC57-PEDV-S plasmid was used as a template.

To improve the detection sensitivity and reduce the dependence on equipment, we combined RAA with CRISPR/Cas12a-FDS and increased the molarity of the reporter to 100 pmol per reaction, so that the green fluorescence could be observed under UV light with the naked eye (Figure 4A). As expected, the LOD was increased from 10^8^ to 10^2^ copies per reaction (Figure 4B). In addition, the specificity and LOD of visualized RAA-CRISPR/Cas12a-FDS were consistent with that of CRISPR/Cas12a-FDS (Figures 4C–E).

### Establishment of CRISPR/Cas12a-LFS

For rapid detection, we substituted the fluorescence intensity readout of the RAA-CRISPR-FDS with LFSs to establish the CRISPR/Cas12a-LFS (Figure 1A). The optimal reaction conditions identified for CRISPR/Cas12a-FDS were used for CRISPR/Cas12a-LFS to test its reduces specificity. As a practical application, only PEDV/JSX2014 of Genotype II was positive, whereas PEDV/CV777 of Genotype I and other swine viruses were negative, indicating that CRISPR/Cas12a-LFS has good specificity (Figure 1B). Moreover, the LOD of the CRISPR/Cas12a-LFS was 1 × 10^2^ copies of template DNA (Figure 1C).

### Comparative Evaluation of Clinical Sample Detection Using CRISPR/Cas12a-LFS and qRT-PCR

**Figure 5:**
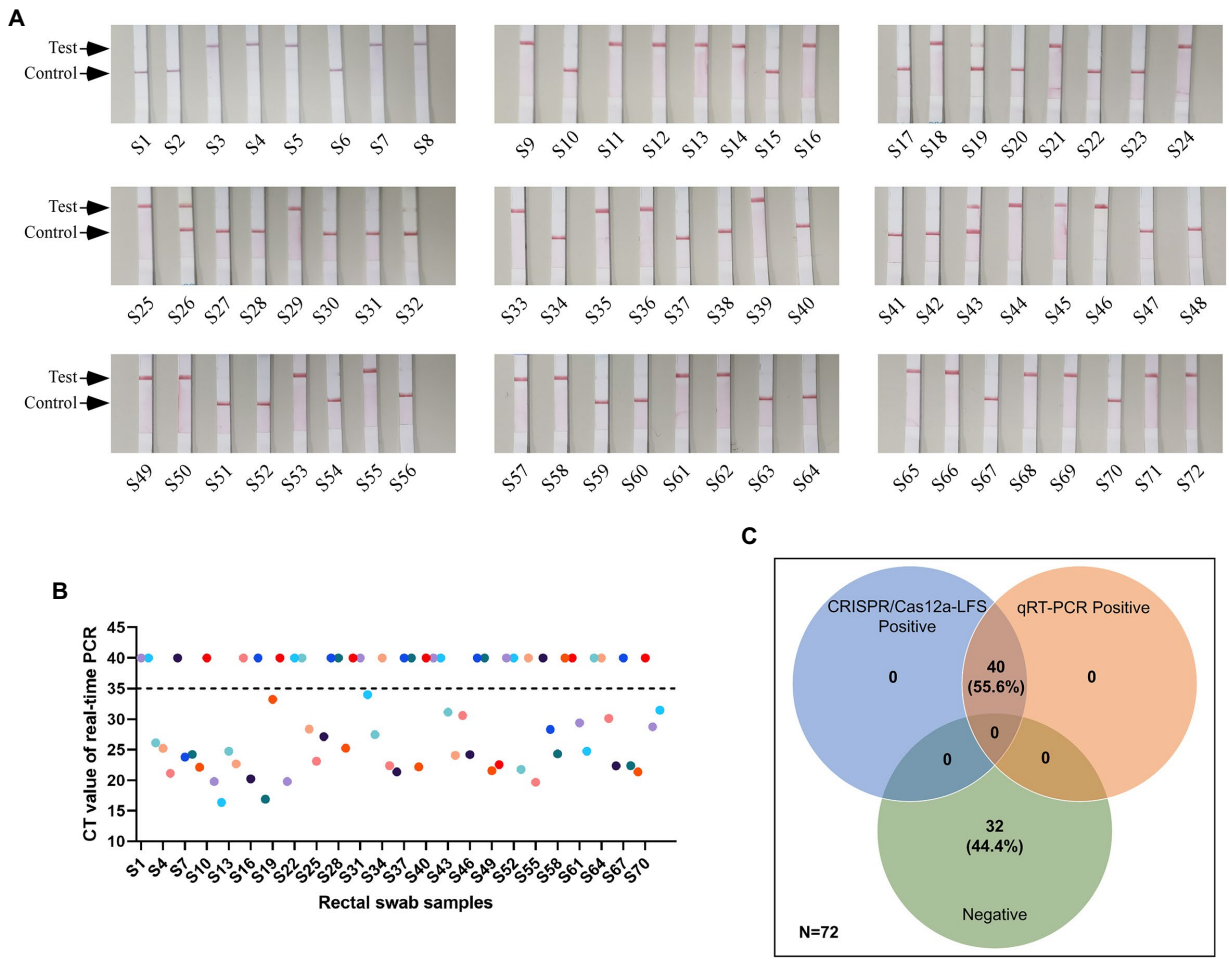
Detection results of clinical samples. **(A)** Detection of PEDV cDNA in 72 rectal swab samples using CRISPR/Cas12a-LFS. The test line and control line on the lateral strip were marked with arrows. **(B)** Detection of PEDV cDNA in 72 rectal swab samples using quantitative real-time PCR (qRT-PCR). **(C)** The Venn diagram shows the consistency between the CRISPR/Cas12a-LFS and qRT-PCR assays.

The anal swabs collected from pigs with diarrhea were used to perform the detection and comparison of CRISPR/Cas12a-LFS and qRT-PCR assays. The positive rate for both methods was 55.8% (Figures 5A,B). Compared with qRT-PCR, CRISPR/Cas12a-LFS also correctly identified and differentiated all 40 positive samples and demonstrated 100% coincidence (Figure 5C).

## Discussion

The emergence of the PED epidemic has caused significant economic losses globally and raised public and animal health concerns (Lin et al., 2016). PED is fatal to unvaccinated or poorly managed pig herds and the vaccine strain of CV777 does not provide sufficient protection against the GII PEDVs currently circulating (Zhang et al., 2020). Early diagnosis is an important for the prevention and control of PED. Given the diagnostic sensitivity and specificity and rapid turnaround time for results, molecular diagnostic assays, such as conventional and real-time reverse transcriptase-PCR (RT-PCR) assays, have become the mainstream methods to detect PEDV (Kim et al., 2007). However, the real-time PCR method is limited by expensive equipment and the need for professional diagnostic laboratories. It also requires significant time for sample transportation from the farm to the laboratory. Therefore, it is important to develop a rapid detection assay for GII PEDV.

Wang et al. (2020) established a rapid, sensitive and instrument-free African swine fever virus (ASFV) detection method based on DETECTR with LFSs (CRISPR/Cas12a-LFS). The entire detection process can be completed in an hour (Wang et al., 2020). Because of the high conservation between the N gene sequences, the detection methods that target the N gene have *a more extensive* detection range compared with the S gene, but cannot distinguish between the GI and GII strains (Miller et al., 2016). Liu et al. (2022) developed a CRISPR-/Cas12a-based detection system combined with multiplex reverse transcriptase loop-mediated isothermal amplification, which allows the detection of PEDV, TGEV, PDCoV, and SADS-CoV with the naked eye. However, they do not perform an experiment to identify different types of PEDV strains. Based on data on the epidemic genotype, we developed a rapid CRISPR/Cas12a-LFS detection assay for GII PEDV. The LOD of the assay is 100 copies/μl and the entire detection process takes approximately 1.5 h.

Compared with the PEDV wild-type strains, there was a 51 nt nucleotide deletion of ORF3 gene in PEDV vaccine strains. The differences in ORF3 may serve as markers to differentiate PEDV attenuated vaccine from wild-type strain (Liu et al., 2019). Yang et al. (2021) established a reverse transcription–enzymatic recombinase amplification method coupled with CRISPR/Cas12a against ORF3 gene, which can specifically detect PEDV variant strains. In the present study, the S gene of the variant and vaccine strains was compared and analyzed. Then, crRNAs were designed on the basis of the results. We established and visualized CRISPR/Cas12a-FDS and CRISPR/Cas12a-LFS assays., which can meet different detection requirement. All of the reaction conditions, reagents, temperatures, and detection equipment are easy to acquire. After RNA extraction, reverse transcription was followed by a 30 min incubation with the addition of nucleic acids to the CRISPR/Cas12a reaction system for readthrough and visualization. This saves more than 1 h of assay time compared with the other nucleic acid detection methods. The CRISPR/Cas12a-FDS also enables kinetic tests on reporter cleavage, providing information for reaction optimization and potentially more accurate detection. Including the time required for RAA and CRISPR/Cas12a detection, the reaction time of the entire CRISPR/Cas12a-LFS was approximately 1.5 h (Figure 1A). The coincidence rate between CRISPR/Cas12a-LFS and qRT-PCR for 72 clinical samples is 100% (Figure 5C). Compared with qRT-PCR, the CRISPR/Cas12a detection platform is a more promising option for on-site detection, because it is free of the limitations of large-scale instruments, expertise and requires a shorter detection time.

In conclusion, we developed a simple, economical, portable and highly sensitive detection platform for GII PEDV. This assay can determine whether pigs should be immunized with PEDV/CV777 vaccine or not, avoiding the false-positive results of the attenuated vaccine strain, PEDV/CV777. It will contribute to the prevention and control of PEDV mutants that have appeared worldwide.

## Data Availability Statement

The raw data supporting the conclusions of this article will be made available by the authors, without undue reservation. We have uploaded our raw data to Figshare, the website is “https://doi.org/10.6084/m9.figshare.19792942.v1”.

## Author Contributions

FX and BQ conceived and designed the project. BQ, KL, DZ, and WP performed the most experiments with assistance from YW and YC. WN and YH analyzed the data. BQ and KL wrote the manuscript with review comments from all authors. XW, JL, and ZB edited the paper. FX, XZ, and JD supervised and managed the project. All authors contributed to the article and approved the submitted version.

## Funding

This study was funded by the National Key R&D Program (2021YFD1800500), Jiangsu Agricultural Science and Technology Innovation Fund [CX(21)2038], and the Guidance Foundation, the Sanya Institute of Nanjing Agricultural University Research (NAUSY-ZD08).

## Conflict of Interest

The authors declare that the research was conducted in the absence of any commercial or financial relationships that could be construed as a potential conflict of interest.

## Publisher’s Note

All claims expressed in this article are solely those of the authors and do not necessarily represent those of their affiliated organizations, or those of the publisher, the editors and the reviewers. Any product that may be evaluated in this article, or claim that may be made by its manufacturer, is not guaranteed or endorsed by the publisher.

## References

[ref1] Chasey D. Cartwright S. F. (1978). Virus-like particles associated with porcine epidemic diarrhoea. Res. Vet. Sci. 25, 255–256. doi: 10.1016/S0034-5288(18)32994-1, PMID: 103154 PMC7130664

[ref2] Chen Q. Li G. Stasko J. Thomas J. T. Stensland W. R. Pillatzki A. E. . (2014). Isolation and characterization of porcine epidemic diarrhea viruses associated with the 2013 disease outbreak among swine in the United States. J. Clin. Microbiol. 52, 234–243. doi: 10.1128/JCM.02820-13, PMID: 24197882 PMC3911415

[ref3] Chen J. S. Ma E. Harrington L. B. Da Costa M. Tian X. Palefsky J. M. . (2018). CRISPR-Cas12a target binding unleashes indiscriminate single-stranded DNase activity. Science 360, 436–439. doi: 10.1126/science.aar6245, PMID: 29449511 PMC6628903

[ref4] Gootenberg J. S. Abudayyeh O. O. Kellner M. J. Joung J. Collins J. J. Zhang F. (2018). Multiplexed and portable nucleic acid detection platform with Cas13, Cas12a, and Csm6. Science 360, 439–444. doi: 10.1126/science.aaq0179, PMID: 29449508 PMC5961727

[ref5] Gootenberg J. S. Abudayyeh O. O. Lee J. W. Essletzbichler P. Dy A. J. Joung J. . (2017). Nucleic acid detection with CRISPR-Cas13a/C2c2. Science 356, 438–442. doi: 10.1126/science.aam9321, PMID: 28408723 PMC5526198

[ref6] Huang Y. W. Dickerman A. W. Piñeyro P. Li L. Fang L. Kiehne R. . (2013). Origin, evolution, and genotyping of emergent porcine epidemic diarrhea virus strains in the United States. MBio 4, e00737–e00713. doi: 10.1128/mBio.00737-13, PMID: 24129257 PMC3812708

[ref7] Jinek M. Chylinski K. Fonfara I. Hauer M. Doudna J. A. Charpentier E. (2012). A programmable dual-RNA-guided DNA endonuclease in adaptive bacterial immunity. Science 337, 816–821. doi: 10.1126/science.1225829, PMID: 22745249 PMC6286148

[ref8] Jung K. Saif L. J. (2015). Porcine epidemic diarrhea virus infection: etiology, epidemiology, pathogenesis and immunoprophylaxis. Vet. J. 204, 134–143. doi: 10.1016/j.tvjl.2015.02.017, PMID: 25841898 PMC7110711

[ref9] Kim S. H. Kim I. J. Pyo H. M. Tark D. S. Song J. Y. Hyun B. H. (2007). Multiplex real-time RT-PCR for the simultaneous detection and quantification of transmissible gastroenteritis virus and porcine epidemic diarrhea virus. J. Virol. Methods 146, 172–177. doi: 10.1016/j.jviromet.2007.06.021, PMID: 17697717 PMC7119650

[ref10] Li W. Li H. Liu Y. Pan Y. Deng F. Song Y. . (2012). New variants of porcine epidemic diarrhea virus, China, 2011. Emerg. Infect. Dis. 18, 1350–1353. doi: 10.3201/eid1803.120002, PMID: 22840964 PMC3414035

[ref11] Li W. Van Kuppeveld F. J. M. He Q. Rottier P. J. M. Bosch B. J. (2016). Cellular entry of the porcine epidemic diarrhea virus. Virus Res. 226, 117–127. doi: 10.1016/j.virusres.2016.05.031, PMID: 27317167 PMC7114534

[ref12] Li Y. Wang G. Wang J. Man K. Yang Q. (2017). Cell attenuated porcine epidemic diarrhea virus strain Zhejiang08 provides effective immune protection attributed to dendritic cell stimulation. Vaccine 35, 7033–7041. doi: 10.1016/j.vaccine.2017.10.052, PMID: 29100707 PMC7115645

[ref13] Lin C. M. Saif L. J. Marthaler D. Wang Q. (2016). Evolution, antigenicity and pathogenicity of global porcine epidemic diarrhea virus strains. Virus Res. 226, 20–39. doi: 10.1016/j.virusres.2016.05.023, PMID: 27288724 PMC7111424

[ref14] Liu J. Li L. M. Han J. Q. Sun T. R. Zhao X. Xu R. T. . (2019). A TaqMan probe-based real-time PCR to differentiate porcine epidemic diarrhea virus virulent strains from attenuated vaccine strains. Mol. Cell. Probes 45, 37–42. doi: 10.1016/j.mcp.2019.04.003, PMID: 31004698

[ref15] Liu J. Tao D. Chen X. Shen L. Zhu L. Xu B. . (2022). Detection of four porcine enteric coronaviruses using CRISPR-Cas12a combined with multiplex reverse transcriptase loop-mediated isothermal amplification assay. Viruses 14:833. doi: 10.3390/v14040833, PMID: 35458562 PMC9032155

[ref16] Makarova K. S. Zhang F. Koonin E. V. (2017a). SnapShot: class 1 CRISPR-Cas systems. Cell 168, 946–946.e1. doi: 10.1016/j.cell.2017.02.018, PMID: 28235204

[ref17] Makarova K. S. Zhang F. Koonin E. V. (2017b). SnapShot: class 2 CRISPR-Cas systems. Cell 168, 328–328.e1. doi: 10.1016/j.cell.2016.12.038, PMID: 28086097

[ref18] Mali P. Yang L. Esvelt K. M. Aach J. Guell M. Dicarlo J. E. . (2013). RNA-guided human genome engineering via Cas9. Science 339, 823–826. doi: 10.1126/science.1232033, PMID: 23287722 PMC3712628

[ref19] Miller L. C. Crawford K. K. Lager K. M. Kellner S. G. Brockmeier S. L. (2016). Evaluation of two real-time polymerase chain reaction assays for porcine epidemic diarrhea virus (PEDV) to assess PEDV transmission in growing pigs. J. Vet. Diagn. Investig. 28, 20–29. doi: 10.1177/1040638715621949, PMID: 26699519

[ref20] Pan Z. Lu J. Wang N. He W. T. Zhang L. Zhao W. . (2020). Development of a TaqMan-probe-based multiplex real-time PCR for the simultaneous detection of emerging and reemerging swine coronaviruses. Virulence 11, 707–718. doi: 10.1080/21505594.2020.1771980, PMID: 32490723 PMC7549975

[ref21] Shmakov S. Smargon A. Scott D. Cox D. Pyzocha N. Yan W. . (2017). Diversity and evolution of class 2 CRISPR-Cas systems. Nat. Rev. Microbiol. 15, 169–182. doi: 10.1038/nrmicro.2016.184, PMID: 28111461 PMC5851899

[ref22] Song D. Moon H. Kang B. (2015). Porcine epidemic diarrhea: a review of current epidemiology and available vaccines. Clin. Exp. Vaccine Res. 4, 166–176. doi: 10.7774/cevr.2015.4.2.166, PMID: 26273575 PMC4524901

[ref23] Su S. Wong G. Shi W. Liu J. Lai A. C. K. Zhou J. . (2016). Epidemiology, genetic recombination, and pathogenesis of coronaviruses. Trends Microbiol. 24, 490–502. doi: 10.1016/j.tim.2016.03.003, PMID: 27012512 PMC7125511

[ref24] Sun R. Q. Cai R. J. Chen Y. Q. Liang P. S. Chen D. K. Song C. X. (2012). Outbreak of porcine epidemic diarrhea in suckling piglets, China. Emerg. Infect. Dis. 18, 161–163. doi: 10.3201/eid1801.111259, PMID: 22261231 PMC3381683

[ref25] Vlasova A. N. Marthaler D. Wang Q. Culhane M. R. Rossow K. D. Rovira A. . (2014). Distinct characteristics and complex evolution of PEDV strains, North America, may 2013-February 2014. Emerg. Infect. Dis. 20, 1620–1628. doi: 10.3201/eid2010.140491, PMID: 25279722 PMC4193278

[ref26] Wang X. Ji P. Fan H. Dang L. Wan W. Liu S. . (2020). CRISPR/Cas12a technology combined with immunochromatographic strips for portable detection of African swine fever virus. Commun. Biol. 3:62. doi: 10.1038/s42003-020-0796-5, PMID: 32047240 PMC7012833

[ref27] Yang K. Liang Y. Li Y. Liu Q. Zhang W. Yin D. . (2021). Reverse transcription-enzymatic recombinase amplification coupled with CRISPR-Cas12a for rapid detection and differentiation of PEDV wild-type strains and attenuated vaccine strains. Anal. Bioanal. Chem. 413, 7521–7529. doi: 10.1007/s00216-021-03716-7, PMID: 34686895 PMC8536470

[ref28] Zhang Y. Chen Y. Yuan W. Peng Q. Zhang F. Ye Y. . (2020). Evaluation of cross-protection between G1a- and G2a-genotype porcine epidemic diarrhea viruses in suckling piglets. Animals 10:1674. doi: 10.3390/ani10091674, PMID: 32957461 PMC7552732

[ref29] Zhao P. D. Bai J. Jiang P. Tang T. S. Li Y. Tan C. . (2014). Development of a multiplex TaqMan probe-based real-time PCR for discrimination of variant and classical porcine epidemic diarrhea virus. J. Virol. Methods 206, 150–155. doi: 10.1016/j.jviromet.2014.06.006, PMID: 24928691

[ref30] Zheng L. L. Cui J. T. Han H. Y. Hou H. L. Wang L. Liu F. . (2020). Development of a duplex SYBR GreenI based real-time PCR assay for detection of porcine epidemic diarrhea virus and porcine bocavirus3/4/5. Mol. Cell. Probes 51:101544. doi: 10.1016/j.mcp.2020.101544, PMID: 32109535

