# Supplementary material for: Clustered Regularly Interspaced Short Palindromic Repeat/Cas12a Mediated Multiplexable and Portable Detection Platform for GII Genotype Porcine Epidemic Diarrhoea Virus Rapid Diagnosis
Source: Front Microbiol. 2022 Jun 9;13:920801. doi: 10.3389/fmicb.2022.920801 (PMC9218691; doi:10.3389/fmicb.2022.920801)
Supplement: Supplementary file 1 [file Table_1.DOCX]

**Supplementary Table 1** Primers and probe used in this study

| **Primer name** | **Primer sequence (5ʹ to 3ʹ)** |
| --- | --- |
| PEDV-S-F | CCGGAATTC ATGAAGTCTTTAACCTACTTCTGGT |
| PEDV-S-R | CGCGGATCC TCACTGCACGTGGACC |
| PEDV-RAA-F | TACTTCTGGTTGTTCTTACCAGTACTTTCA |
| PEDV-RAA-R | CTTATGTAAATAAAGCTGGTAACCACTAGG |
| PEDV-q-F | GTTGTACTGGGCGGTTATCT |
| PEDV-q-R | CCATGAACGCCACTAGCAGT |
| PEDV-probe | VIC-TGGTACTGTGCTGGCCAACATCCA-BHQ1 |
| ssDNA-FQ-reporter | FAM-NNNNNNNNNNNN-Quencher |
| ssDNA-FD-reporter | FAM-NNNNNNNNNNNN-Digoxin |

**Supplementary Table 2** crRNAs targeting PEDV Spike gene

| **crRNA name** | **Sequence (5ʹ to 3ʹ)** | **Direction** |
| --- | --- | --- |
| crRNA1 | *TTTC* ACCAATAGGTAGATAACCGCC | *-* |
| crRNA2 | *TTTG* TTAGCCATATTAGAGGTGGTC | *+* |
| crRNA3 | *TTTC* CTAGCATTAAAACATTGGGCC | *+* |
| crRNA4 | *TTTG* CTACTGAGCCCAATGGCCACA | *+* |
| crRNA5 | *TTTA* ATCAAACGATCGATGGTGTTT | *+* |

Note: The protospacer adjacent motif (PAM) of crRNA recognize in italic
